# K-*ras *mutations in sinonasal cancers in relation to wood dust exposure

**DOI:** 10.1186/1471-2407-8-53

**Published:** 2008-02-20

**Authors:** Jette Bornholdt, Johnni Hansen, Torben Steiniche, Michael Dictor, Annemarie Antonsen, Henrik Wolff, Vivi Schlünssen, Reetta Holmila, Danièle Luce, Ulla Vogel, Kirsti Husgafvel-Pursiainen, Håkan Wallin

**Affiliations:** 1 National Research Centre for the Working Environment, Lersø Parkallé 105, DK-2100 Copenhagen, Denmark; 2 Danish Cancer Society, Institute of Cancer Epidemiology, Strandboulevarden 49, DK-2100 Copenhagen, Denmark; 3 Vejle Hospital, Department of pathology, Kabbeltoft 25, 7100 Vejle, Denmark; 4 Lund University Hospital, Department of Pathology, Sölvegatan 25, SE-221 85 Lund, Sweden; 5 Roskilde Hospital, Department of Pathology, Køgevej 7-13, DK-4000 Roskilde, Denmark; 6 Finnish Institute of Occupational Health, Topeliuksenkatu 41aA, 00250 Helsinki, Finland; 7 Department of Environmental and Occupational Medicine, Aarhus, Vennelyst Boulevard 6, DK-8000 Aarhus C, Denmark; 8 Inserm U88, Saint-Maurice, Hôpital National de Saint- Maurice, 14, rue du Val d'Osne 94415 Saint-Maurice Cedex, France

## Abstract

**Background:**

Cancer in the sinonasal tract is rare, but persons who have been occupationally exposed to wood dust have a substantially increased risk. It has been estimated that approximately 3.6 million workers are exposed to inhalable wood dust in EU. In previous small studies of this cancer, *ras *mutations were suggested to be related to wood dust exposure, but these studies were too limited to detect statistically significant associations.

**Methods:**

We examined 174 cases of sinonasal cancer diagnosed in Denmark in the period from 1991 to 2001. To ensure uniformity, all histological diagnoses were carefully reviewed pathologically before inclusion. Paraffin embedded tumour samples from 58 adenocarcinomas, 109 squamous cell carcinomas and 7 other carcinomas were analysed for K-*ras *codon 12, 13 and 61 point mutations by restriction fragment length polymorphisms and direct sequencing. Information on occupational exposure to wood dust and to potential confounders was obtained from telephone interviews and from registry data.

**Results:**

Among the patients in this study, exposure to wood dust was associated with a 21-fold increased risk of having an adenocarcinoma than a squamous cell carcinoma compared to unexposed [OR = 21.0, CI = 8.0–55.0]. K-*ras *was mutated in 13% of the adenocarcinomas (seven patients) and in 1% of squamous cell carcinomas (one patient). Of these eight mutations, five mutations were located in the codon 12. The exact sequence change of remaining three could not be identified unambiguously. Among the five identified mutations, the G→A transition was the most common, and it was present in tumour tissue from two wood dust exposed adenocarcinoma patients and one patient with unknown exposure. Previously published studies of sinonasal cancer also identify the GGT → GAT transition as the most common and often related to wood dust exposure.

**Conclusion:**

Patients exposed to wood dust seemed more likely to develop adenocarcinoma compared to squamous cell carcinomas. K-*ras *mutations were detected in 13% of adenocarcinomas. In this study and previously published studies of sinonasal cancer the found K-*ras *mutations, were almost exclusively G → A transitions. In conclusion, our study, based on a large representative collection of human SNC tumours, indicates that K-*ras *mutations are relatively infrequent, and most commonly occur in adenocarcinomas. Wood dust exposure alone was not found to be explanatory for the G→A mutations, but combination of exposure to tobacco, wood dust, and possibly other occupational agents may be a more likely explanation. Overall, the study suggests a limited role for K-*ras *mutations in development of sinonasal cancer.

## Background

Cancer of the sinonasal cavities is rare with an annual standardized incidence rate of 0.46 per 100 000 person-years for men and 0.62 for women in Denmark year 2001 [1]. In 1967, Acheson et al. reported of a cluster of patients with sinonasal adenocarcinomas in the furniture industry in Buckinghamshire, Great Britain [2]. Since then, several studies have found an association between exposure to wood dust and sinonasal cancer [3-5]. In particular, adenocarcinoma has been strongly associated with exposure to hardwood dust [6]. In a recent reanalysis of 12 pooled studies, an odds ratio of 45.5 [CI = 28.3–72.9] for adenocarcinoma was found among male workers occupied in jobs with high levels of wood dust exposure [7]. The International Agency for Research on Cancer (IARC) classified "furniture and cabinet making" as carcinogenic to humans in 1987 and so was wood dust in 1995 [8].

Mutagenic carcinogens can leave fingerprints such as specific mutation patterns, which may help identify them as environmental risk factors [9]. Because tumour suppressor genes and oncogenes are involved in carcinogenesis, they are good candidates for such investigations. One of the most well established links between a pattern of mutation and environmental exposure is ultraviolet light induced mutation in the *p53 *gene. UV-light damages DNA by the formation of dipyrimidine adducts between two adjacent pyrimidines. This adduct is converted to a unique UV induced transition, the CC→TT tandem mutation, which is detected at high frequencies in human skin cancers [10-12]. It is thus a fingerprint of UVB induced DNA damage.

Mutations in the activating codons of *ras *(12, 13 and 61) are detected in 20–30% of all human cancers, varying in frequency between the different organs. E.g. < 90% of pancreas cancers harbour *ras *mutations [13]. Codon 12 is a preferential site for DNA adduct formation for several carcinogens [14]. Smoking may be contributing to sinonasal cancer. Lung cancers from smokers frequently harbours mutations in the *ras *gene codon 12 [15], the most frequent type of mutations is a G•C→T•A transversion in K-*ras *codon 12 [16]. The benzo(a)pyrene diol-epoxide-guanine adduct, that is most frequently produced when benzo(a)pyrene metabolites reacts with DNA, has been identified in the lungs of smokers[17]. This adduct is most frequently transformed into G•C to T•A transversions [17]. The nitrosamine NNK, a tobacco-specific lung carcinogen, is associated with G•C → A•T transitions in rodent lung tumours [18]. Among non-smokers with lung cancer the G•C → A•T transition is the most frequent point mutation [15].

Genetic changes in sinonasal adenocarcinomas have been reported occasionally. Studies including a limited number of patients indicate, that the *ras *genes are mutated in some tumours [19-22]. In the studies of Saber et al [20] and Yom et al [21], K-*ras *was mutated in approximately 14% of the tumours. In the study of Spanish intestinal-type adenocarcinomas, 50% of the tumours harboured mutations [23]. The G•C → A•T transition was among the most common mutations found, but interpretation of the results has been limited due to the small sample sizes. These previous studies indicated that wood dust exposure might be correlated to a specific mutation pattern.

## Methods

### Study subjects and tumour material

This study was reviewed and approved by the Danish National Committee on Biomedical Research Ethics (journal number: KF 01-048/02). Records on potential study subjects with sinonasal cancer were retrieved from the nationwide Danish Cancer Registry. This computerized register, which is regarded as almost complete, uses the unique Central Person Registry Number (CPR) as the key identifier of each patient. This CPR-number is applied to all citizens in Denmark, and is used all over the society for administrative identification of persons. It includes information on name and address, vital status, dates of eventual decease or emigration, and to some extent on relatives. From the start of the Danish Cancer registry in 1942 an extended version of the 7th revised of the International Classification of Disease for classification has been used for classification of cancers. Since 1978 tumours have also been classified according to the International Classification of Disease for Oncology, including detailed information of topography and morphology [1]. By use of a combination of the two classification schemes we selected records of the in total 466 patients born after 1903 and registered with adenocarcinomas or squamous cell carcinomas between 1991 and 2001, with ICD7 code 160.

The inclusion criteria for study were, that the tumour should be located in the nasal cavity or paranasal sinuses, and only the diagnoses listed in Table 1, were included in the study. Among the initial 466 patient retrieved form the cancer registry 11 were excluded because there was unequivocal information on the notifying hospital and the patients was not treated in the university hospitals, therefore no pathological information could be retrieved. An additional 32 study subjects were excluded because the pathology reports were missing in the hospital archives. For the remaining 423 study subjects we received one or more pathology reports. On the basis of the entire information in these reports, 215 patients were excluded because the diagnoses did not comply with our inclusion criteria. A major part of the excluded tumours was located in the vestibulum nasi or in the nasopharynx. If a report was not accurate enough to include or exclude a study subject, we retrieved paraffin embedded tumour samples (PET) and original sections. These were then evaluated by our pathologists resulting in exclusion of an additional 34 patients. One of these patients was excluded because the tumour block contained less than 10% tumour tissue. The remaining 174 study subjects were included in the present study (Table 1).

**Table 1:** A detailed list of histological diagnoses included in the molecular epidemiology study, with reference to the WHO classification of tumours (WHO, 1991). SNOMED, Systemic Nomenclature of Medicine and the distribution of the histology of the samples.

**WHO code**	**SNOMED 1991**	**Diagnosis**	**Comments**	**Subtype**	**Distribution in the study**
1.2.1	8121/3	Sinonasal carcinoma	SNUC, refer to IHC	Not othervice specified Undifferentiated	6 1
1.2.1.1	8070/3	Squamous cell carcinoma		keratinising and nonkeratinising associated with inverted papilloma	104 3
1.2.1.2	8121/3	Cylindrical cell carcinoma			-
1.2.2	8051/3	Verrucous squamous cell carcinoma			2
1.2.3	8074/3	Spindle cell carcinoma			-
1.2.4	8140/3	Adenocarcinoma		Low grade low grade – acinic like high grade	11 1 22
1.2.5	8260/3	Papillary adenocarcinoma			1
1.2.6	8144/3	Intestinal-type adenocarcinoma			23
1.2.18	8041/3	Small cell carcinoma	Probably metastasis from primary lung tumour		-
1.2.19	8082/3	Lymphoepithelial carcinoma			-
1.2.15	8560/3	Adenosquamous carcinoma			-

### Tissue samples

PET blocks were sectioned serially to prepare material for pathological review and molecular analysis: For the histopathological review, one section was stained with hematoxylin and eosin and one with alcien blue-PAS. Two times five 10-μm sections were collected for DNA extraction. A final section was cut for hematoxylin and eosin staining to confirm that there was tumour tissue in the intervening sections. On review the ratio of tumour cells to non-malignant cells in the tissue sections was estimated. In a few cases additional sections were cut for automated immunohistochemical staining.

### Review of the histological diagnoses

To ensure uniform tumour classification according to modern criteria, the slides were coded and histological diagnoses were evaluated independently by two of us (M.D. and T.S) without knowledge of the original diagnosis. Both reviewers assigned a concordant diagnosis in almost all cases. For a few in which there was disagreement, both reviewers re-examined the slides in concert to reach consensus. In seven cases, immunohistochemical staining was performed to determine the final diagnosis.

### Exposure to wood dust and potential confounders

Three different sources were used to obtain information on exposure to wood dust and other potential risk factors. First, all study subjects were retrieved based on the CPR-number in the computerized National Pension Fund, for which membership has been compulsory for all wage earners in Denmark since the start of this scheme in 1964. This register keeps historical information on all companies in Denmark. If a person has been an employee, a working history stating dates of start and end of each employment can be retrieved. Records have been kept even when a person has retired or deceased. Further, each company has by Statistics Denmark been classified according to an extended version of International Standard Industrial Classification of all economic activities, which includes detailed information on trades where exposure to wood dust is normal, e.g. wood furniture industry or carpentry shops, etc. Based on this information we reconstructed the employment- and potential wood dust exposure history from 1964 and until diagnosis. We also extracted a job title from the Central Person Registry and classified this according to potential wood dust exposure. Finally, we obtained information on occupational exposure to risk factors for sinonasal carcinomas including wood dust, tobacco smoking, formaldehyde, chromium(VI), nickel, textile and leather dust by a structured telephone interview either with the patient or if he or she was deceased with a next-of-kin, preferable the last spouse or a child. Information on vital status, relatives and contact addresses was obtained from the CPR-registry. The CPR-registry, which we used to identify contact addresses on patients, their spouses and children, was established in 1968 and included all persons alive from the first of April 1968 until today. Family relations were, at the onset of the CPR register presumed to be the persons living at the same address. Therefore, for the link between parents and their children, the CPR register is only regarded complete for persons born in 1930 and onwards. However, information before 1930 still exists in the registry, but is of more sporadic character. Since, the majority of our cases with sinonasal cancer (n = 103) was born before 1930, and had already died at the onset of this study, cases for whom we could identify relatives to contact, were primarily limited to those born after 1930. Among the 103 patients born before 1930, a contact person could only be identified for 31 patients (25 of these completed an interview). For the 71 patients born after 1930 a contact person could be identified for 67 persons (60 of these completed an interview). In total 85 interviews were successfully completed.

### DNA purification

Genomic DNA was prepared from five 10-μm sections. The sections were extracted twice with 1 ml xylene (Merck) and twice with 1 ml 96% ethanol, each for 10 min at 55°C with centrifugation in between. After the last wash with ethanol, the tissue was dried and rehydrated with 500 μl lysis buffer (50 mM Tris-HCl pH 8.5, 1.0 mM EDTA, 0.5% Tween-20) for 2 hours (55°C) under rotation. The tissue was agitated with 50–100 μl 20 mg/ml proteinase K (Finnzymes, Finland) depending on sample size, overnight or until the tissue was totally degraded. The samples were extracted twice with 500 μl phenol/chloroform/water mixture (Applied Biosystems, cat. no 400765) and once with 500 μl chloroform (AMRESCO, Ohio, US). The DNA was precipitated for 2 hours at -20°C with 27 μl 3 M sodium acetate in ice-cold ethanol. The DNA was washed once with 70% ethanol, and dried at 50°C before it was dissolved in RNase free water. The DNA concentration was determined spectrophotometrically at 260 and 280 nm, and where possible, the concentration was adjusted to 100 μg/ml.

### RFLP analyses

In order to identify mutations in the K-*ras *gene codon 12, codon 13 and codon 61, three assays were developed. The assays were modified versions of the methods used by Saber et al [20] and Vachtenheim et al [24]. Separate PCR reactions were performed for the three assays. The reactions were performed with the primers listed in Table 2 under the conditions listed in Table 3, the amount of DNA varied from 0.5 μl to 4 μl since individual optimization for each sample was necessary.

**Table 2:** Primers and restriction enzymes used in the RFLP assays.

Exon	Codon	Primers	Sequence 5' to 3'	Restriction enzymes/recognition site	PCR product/cut fragments
1	12	RFLP12F	ACT GAA TAT AAA CTT GTG GTA GTT GGA **C**CT	BstN I : 5'..CCTGG..3' 3'..GGACC..5'	157 bp/ N: 114+29+14 M:143+14
		RFLP1213R	TCA AAG AAT GGT CCT G**G**A CC		
					
1	13	RFLP13F	ATA TAA ACT TGT GGT AGT T**CC** AGC TGG	Van 91 I : 5'.CCA(N)_5_TGG.3' 3'.GGT(N)_5_ACC.5'	152 bp/ N:126+26 M:152
		RFLP1213R	TCA AAG AAT GGT CCT G**G**A CC		
					
2	61	RFLP61F	CTT GGA TAT TCT CGA CAC AGC **T**G**A** T	Bcl I: 5'..TGATCA..3' 3'..ACTAGT..5'	179 bp/ N:155 M: 179
		RFLP61R	AAC TAT AAT TAC TCC TTA ATG TCA GCT TA		

**Table 3:** Reaction conditions for all the PCR reactions.

Assay\PCR conditions	KCl	Tris-HCl	MgCl_2_	dNTP	Taq polymerase	Primers _forward + reverse_	pH
RFLP12	50 mM	20 nM	0.25 mM	0.2 mM	0.75 unit	0.5 μM	8.4
RFLP13	50 mM	20 nM	0.25 mM	0.2 mM	0.75 unit	0.5 μM	8.4
RFLP61	50 mM	10 nM	1.5 mM	0.2 mM	1.0 unit	1.0 μM	8.3
SEQ1213	50 mM	10 nM	1.5 mM	0.2 mM	1.0 unit	1.0 μM	9.0
SEQ61	50 mM	10 nM	1.5 mM	0.2 mM	1.0 unit	1.0 μM	9.0

The PCR reactions were carried out on a PTC-100™ Programmable Thermal Controller (MJ Research, Inc). The cycling conditions for the RFLP12 assay and the RFLP13 assay were; denaturation at 94°C for 1 min, taken though 35 cycles at 94°C for 30 sec, 55°C for 45 sec, 72°C for 45 sec and a final incubation at 72°C for 10 min. For the RFLP61 assay the cycling conditions were: DNA was denatured at 94°C for 2 min, the reactions were taken through 12 cycles of 94°C for 30 sec and 67°C for 1 min with a decrement of -1°C for each cycle (67°C to 56°C), then taken through 28 cycles of 94°C for 40 sec, 56°C for 1 min and finally incubated at 72°C for 10 minutes. For all PCR reactions a no template control was included. All PCR products were visualised on a 1.5% NuSieve^® ^3:1 agarose gel, and only samples where a distinct band of the correct size was observed were used for restriction enzyme digestion.

Before restriction enzyme digestion, the PCR products for the RFLP13 assay were purified by GFX PCR DNA and Gel Band Purification Kit (Amersham Biosciences, UK). The restriction enzyme digestion of the RFLP12 assay was performed with 10 μl PCR product, 1× NEBuffer 2, 100 μg/ml BSA and 10 units of restriction endonuclease BstN1 I (Biolabs, New England), incubated overnight at 60°C. The digestion of the RFLP13 assay was performed in a total volume of 15 μl with 1× Surecut buffer B and 2 units of restriction endonuclease Van 91 I (Boeringer Manheim) with 6 hr of incubation at 37°C. The RFLP61 assay was digested in a total volume of 11.6 μl with 1× NEBuffer 2 and 6 units of restriction endonuclease Bcl I (Biolabs, New England) and 6 hr incubation at 50°C. The digested PCR fragments were visualised on a 3% NuSieve^® ^3:1 agarose gel, run in 1× TBE buffer.

To ensure that we were able to distinguish between a mutant sequence and wild type sequence, we included a negative and a positive control in all digestions. As a negative control, DNA from a healthy person was used. For positive controls in the codon 12 assay the A549 cell line, which contains a homozygous GGT→ AGT mutation was used [25]. For codon 13 the MDA-MB231 cell line with a heterozygous GGC→GAT mutation [26] was used and for codon 61 DNA from a cancer patient with a heterozygous CAA→ CTA mutation was used. To determine the sensitivity of the assays, mutated DNA was diluted with wild type DNA. In the assay RFLP12, a distinct band of mutated PCR products was detected in a mixture containing 6.25 % homozygote DNA which corresponds to 12.5 % tumour tissue assuming a dominant mutation. In the other two assays, the RFLP13 and RFLP61, bands of mutated PCR product were detected with 12.5% mutated heterozygous DNA. A clear cut detection of mutations was therefore possible in samples with a tumour content of 10 % and above in the RFLP assays. Using direct sequencing, a mutation could be detected in samples containing 12.5 % mutated DNA which corresponds to 25 % tumour tissue heterozygous for mutation in a sample (data not shown).

### Direct sequencing

In order to identify the mutations by direct sequencing, two assays were developed, the SEQ1213, SEQ61. In order to generate PCR products for sequencing the following primers were developed; forward1213 5' AAC CTT ATG TGT GAC ATG TTC'3, reverse1213 5' ATG GTC CTG CAC CAG TAA T'3, forward61 5' AAA GGT GCA CTG TAA TAA TCC'3 and reverse61 5' TGA TTT AGT ATT ATT TAT GGC AAA'3. The PCR conditions are listed in Table 3. The PCR reactions were performed in 50 μl reactions, on a PTC-100™ Programmable Thermal Controller (MJ Research, Inc). For the SEQ1213 assay the DNA was denatured at 94°C for 1 min, and amplified in 12 cycles of 94°C for 30 sec and 57°C for 1 min with decrement of -1°C for each cycle (57°C to 45°C). It was amplified through 28 cycles of 94°C for 40 sec, 45°C for 1 min and incubated for 72°C for 10 minutes. For the SEQ61 assay the amplification was done at: 95°C for 2 min then 40 cycles of 94°C for 1 min, 48°C for 30 sec and 72°C for 1 min. For all PCR reactions a control without template was included. The PCR products (3.0 μl) were visualised by electrophoresis, on a 1.5% NuSieve^® ^3:1 agarose gel, run in 1×TBE buffer. Only PCR reactions where a distinct band in the right size appeared were purified using the GFX PCR DNA and Gel Band Purification Kit (Amersham Biosciences, UK) according to the manufacturer. To generate the sequencing templates for the SEQ1213 and 61 the following primers were used; sequence1213 5' ATA TAG TCA CAT TTT CAT TAT T'3 and sequence61 5'TGT TTC TCC CTT CTC AGG ATT C'3 with the BigDye^® ^Terminator Ready Reaction Kit (Applied Biosystems, part no 4303152) according to the manufacturer. The annealing temperatures were SEQ1213, 45°C and SEQ61, 50°C. The sequencing was performed on an ABI 310 Genetic Analyser, according to standard protocols. In order to confirm a mutation, both strands were sequenced a second time from an independent PCR reaction.

### Statistical analysis

In this case-case study we compared historical occupational exposure to wood dust in cases with different histological types of sinonasal cancers, using squamous cell carcinoma cases as a reference group. We also compared this exposure to wood dust of the cases with and without K-*ras *mutations. To estimate the magnitude of the associations, multivariate adjusted odds ratios and their corresponding 95% confidence intervals were calculated by unconditional logistic regression with the statistical package Stata, version 9. Age and sex were included in all models as potential confounders. In the subgroup of 85 cases for which we have individual information on tobacco smoking and occupational exposure to formaldehyde, these were further included in the models. None of the cases with tumours with K-*ras *mutations were exposed to other potential confounders, i.e. leather and textile dust, nickel and chromium(VI).

## Results

### Histology and wood dust exposure

The histological diagnoses included in the study and the number of patients in each category is listed in Table 1. The study included 58 adenocarcinomas, 109 squamous cell carcinomas and 7 carcinomas of other types. A compilation of our assessment is listed in Table 1. Based on our criteria for interview we contacted 97 patients by mail. Interview of the patients were completed for 85 patients (34 adenocarcinomas, 48 squamous cell carcinomas and 3 with other histology). Seven patients refrained from participating in the study (5 with adenocarcinomas and 2 with squamous cell carcinomas) (Table 4). The last 6 patients could not be reached. In the interviews, 65% of the adenocarcinoma patients (22 patients) reported exposure to wood dust. In the squamous cell carcinoma group only 15 % (7 patients) reported that they had been exposed to wood dust. When we evaluated all available exposure data, we concluded that totally 40 patients (23%) had been exposed to wood dust, none of these being women. Thirty-three of the wood dust exposed patients had been diagnosed with adenocarcinomas and seven with squamous cell carcinomas. The frequency of wood dust exposure was 8.9 times more common in the group of adenocarcinomas (33 out of 58 patients were exposed to wood dust) compared to squamous cell carcinomas (7 out of 109 patients were exposed to wood dust). Among our patients exposure to wood dust was associated with a 21-fold [CI = 8.0–55.0] increased risk of having an adenocarcinoma than a squamous cell carcinoma compared to patients not being exposed, when adjusting for age. For those 85 patients that had been interviewed it was possible to investigate the influence of smoking and occupational confounders. Information on both smoking and formaldehyde was available for only 82 of the interviewed patients. In this limited group of patients wood dust exposure was associated with a 47-fold [CI = 7.7–328] increased risk of adenocarcinomas than squamous cell carcinomas compared to unexposed cases, when adjusting for age, tobacco smoking and formaldehyde exposure (Table 5).

**Table 4:** Distribution of exposure to wood dust in relation to histological diagnosis of sinonasal cancers.

Wood dust exposure crude	Males			Females		
	Squamous cell carcinoma	Adenocarcinoma	Other	Squamous cell carcinoma	Adenocarcinoma	Other
No exposure (interview)	19	3	1	22	2	2
No exposure according to registers	32	12	4	29	8	0
Wood dust exposure confirmed by interview	6	29	0	-	-	-
Wood dust exposure according to registers	1	4	0	-	-	-

Total	58	48	5	51	10	2

The wood dust exposure is based on information from three sources: 1, personal interview, 2, trade and job codes from the National Pension Fund (NPF) and 3, self reported job titles in Central Person Registry (CPR). A minimum employment length of one year was set as a lower limit. If a patient's data from NPF or CPR did not imply any wood dust related employments the patient was considered not exposed to wood dust. Patients without any records in NPF and CPR are also regarded as not exposed since they have not been an employee and therefore unlikely to be exposed to wood dust. The information from the interview was weighted more important than that in the data retrieved from the two registers. The patients in the rows of no exposure confirmed by interview and no exposure according to registers are referred to as not exposed to wood dust. The patients in the rows wood dust exposure confirmed by interview and wood dust exposure according to registers are categorized as exposed to wood dust.

**Table 5:** Exposure to wood dust by cases with squamous cell carcinomas and adenocarcinomas

Histology	Wood dust Yes/no	OR^1 ^(95%CI)	OR^2 ^(95%CI)	OR^3 ^(95%CI)
Squamous cell carcinomas	7/102	1	1	1
Adenocarcinomas	33/25	19.2 (7.6–48.5)	21.0 (8.0–55.0)	46.9 (7.7–328)

^1) ^Unadjusted

^2) ^adjusted for age

^3) ^adjusted for age, formaldehyde and tobacco smoking, but data limited to the subgroup of patients (n = 82) for which an interview was completed and information on tobacco smoking and formaldehyde was present (squamous cell: wood dust exposed = 6, not exposed = 41; adenocarcinomas: exposed = 29, not exposed = 6)

### Genetic alterations

The analysis of K-*ras *codon 12, mutations was successful with at least one of the assays for 166 of the samples (95%), whereas for the rest of the samples, no PCR product could be amplified despite several attempts. For codon 13, the success rate was 90 % (157 samples). For codon 61, only the adenocarcinoma samples were analysed by RFLP, with a success rate of 88%. However, direct sequencing was performed on all samples, with a success rate on 91%. Only mutations in codon 12 and 13 were found. The mutations and exposure data for these patients are presented in Table 6.

**Table 6:** Lists the histology, exposure data and mutations from sinonasal patients harbouring K-*ras *mutations.

Patient	Most recent job title according to the Central person registry	Histology	Interview	Smoking	Other potential confunders	Wood dust exposure according to interview	Mutation
15	-	High-grade Adenocarcinoma	No	NA	NA	No	G**G**T→G**A**T
64	Master joiner	Low-grade Adenocarcinoma	Yes	No	Formaldehyde	Yes	Ambiguous (codon 12 3^rd ^or codon 13, nt 1 or 2)
76	Master joiner	Intestinal-type Adenocarcinoma	Yes	Yes	Formaldehyde	Yes	Ambiguous (GGC→G**A**C)
86	Master joiner	Low-grade Adenocarcinoma	Yes	Yes	Formaldehyde	Yes	G**G**T→G**A**T
91	Farmer	Intestinal-type Adenocarcinoma	Yes	Yes	No	Yes	G**G**T→G**A**T
93	Long distance driver	Squamos cell carcinoma	Yes	Yes	No	No	G**G**T→G**C**T
160	Worker	Low-grade Adenocarcinoma	Yes	Yes	No	No	Ambiguous (codon 12, nt 1 or 2)
178	Associate professor	Low-grade Adenocarcinoma	Yes	No	No	No	G**G**T→G**T**T

NA = data not available, since no interview was completed. Wood dust exposure is based on information from interview. Where interview data was not available trade and job codes from the National Pension Fund (NPF) or self reported job titles in Central Person Registry (CPR) were used. A minimum employment length of one year was set as a lower limit. Patients without any records in NPF and CPR are also regarded as not exposed since they have not been an employee and therefore unlikely to be exposed to wood dust. The information from the interview was weighted more important than that in the data retrieved from the two registers.

We identified 8 mutations in total. Three of these were ambiguous since a clear-cut sequencing result could not be obtained due to high background. Of the eight mutations, seven occurred in adenocarcinomas, only one occurred in a squamous cell carcinoma. The identified mutations were all located in codon 12. Three were G•C → A•T transitions causing an amino acid change from glycine to aspartic acid. In one patient a G•C → C•G transversion was identified leading to an amino acid change from glycine to alanine and finally, in one patient a G•C → T•A transversion had occurred changing glycine to valine.

The exposure to wood dust was assessed by interviews and data extracted from the Central Person Registry (CPR) and National Pension Fund (NPF). Patient number 15, harbouring a mutation in codon 12 (GGT^GLY ^→ GAT^ASP^) refrained from being interviewed, no job title was registered in the CPR nor was there a record of employment in the pension fund, making it likely that she has been full-time housewife. The RFLP on samples from patient 64, 76 and 160 indicated mutations. However, the sequencing of the samples could not confirm a mutation despite several sequencing attempts. Patient number 64 reported that he had been a furniture worker/construction carpenter for 37 years. Patient number 76 had worked from a young age as a furniture maker for 47 years with only a few interruptions. Patient number 160 was not classified as having been exposed to wood dust, but had several jobs servicing machines, producing plastic ware and cleaning. The patients number 86 and 91 both had a mutation in codon 12 (GGT^GLY ^→ GAT^ASP^). Patient number 86 reported working as a furniture maker for 19 years, before being employed in an administrative job. Patient number 91 worked mainly with farming and had part time employment as a bricklayer (8-years) and a truck driver at a saw mill (12-years). He was classified as being exposed to wood dust. For patient number 93 a codon 12 (GGT^GLY ^→ GCT^ALA^) mutation was observed. The patient's occupational history, of working as a farm helper, trench-digging machine operator and a long distance van driver, did not reflect exposure to wood dust. Tumour tissue from patient number 178 also contained a mutation in codon 12 (GGT^GLY ^→ GTT^VAL^), and he was also classified as not having been exposed to wood dust. The patient reported in the interview had been working as a clerk in the shipping trade and as a higher education teacher. According to the interviews, patients number 76, 86, 9, 93 and 160 were smokers, whereas patients number 64 and 178 were never smokers. Odds ratios for tobacco smoking and formaldehyde adjusted for age and sex are shown in Table 7 for the subgroup of interviewed patients. There was an OR_unadjusted _of 3.6 [CI = 0.9–15.2] for having a K-*ras *mutation among those who had been exposed to wood dust compared to those who had not been exposed. This odds ratio decreased to 2.0 [CI = 0.2–16.5] after adjustment for potential confounders.

**Table 7:** Exposure to wood dust, tobacco smoking and formaldehyde in sinonasal cancer cases with and without K-*ras *mutations.

		K-ras mutations		
Characteristics	Total	Mutation/wild type	OR^1 ^(95%CI)	OR^2 ^(95%CI)
Wood dust exposure				
No	134	4/130	1	1
Yes	40	4/36	3.6 (0.9–15.2)	2.0 (0.2–16.5)
				
Age	174	8/166	0.97 (0.92–1.02)	1.0 (0.9–1.1)
				
Sex				
Male	111	7/104	1	1
Female	63	1/62	0.2 (0.03–2)	0.3 (0.03–3.1)
				
Tobacco smoking				
No	15	2/13	1	1
Yes	69	5/64	0.5 (0.1–2.9)	0.4 (0.1–2.6)
Unknown	90	1/89	0.1 (0.001–0.9)	-
				
Formaldehyde exposure				
No	50	4/46	1	1
Yes	35	3/32	1.1 (0.2–5.1)	0.5 (0.1–3.9)
Unknown	89	1/88	0.1 (0.01–1.2)	-

The table shows the influence on sex, age and exposure to tobacco smoking, and formaldehyde to the odds ratios for having a K-*ras *mutation. Information on exposures was based on interview and registry data. Information on smoking and exposure to formaldehyde could only be obtained from interviewed persons, which explains the number of persons with unknown status. ^1 ^Unadjusted odds ratio, ^2 ^Mutually adjusted odds ratio.

We determined the sensitivity of the assays by diluting mutated DNA with wild type DNA. In the diluted positive controls we were able to detect mutations, corresponding to samples containing 12.5% tumour material harbouring a heterozygous K-*ras *mutation in the RFLP analysis. There was somewhat less sensitivity than with direct sequencing, but mutations could be detected in samples corresponding to 25% tumour tissue. The average amount of cancer tissue in the blocks was 51% with a standard deviation of 24% and a minimum content of 10%.

## Discussion

Cancers of the sinonasal cavities are strongly correlated to exposure to wood dust, in particular adenocarcinoma has been associated with exposure to hardwood dust in epidemiological studies [3-5,7]. In the present study, we found that 23% of the patients had been exposed to wood dust. It has recently been estimated that 3.3% of the employed population in Denmark are exposed to wood dust [27]. In our study exposure to wood dust adjusted for age was associated with a 21.0-fold [CI = 8.0–55.0] increase in the risk of having an adenocarcinoma than a squamous cell carcinoma. The frequency of wood dust exposure among the patients with adenocarcinomas was 8.9 times more common than among the patients with squamous cell carcinomas. Our findings are some what in accordance with a previously published pooled analysis on eight case/control studies by Mannetje et al [28]. In this analysis the frequency of wood dust exposed adenocarcinomas (74.2%) were five times more common than wood dust exposed squamous cell carcinomas (14.7%).

In the present study, mutations in the K-*ras *gene were detected in 13% of the adenocarcinomas and in 1% of the squamous cell carcinomas. The found frequency of K-ras mutations was lower than expected. Due to the low frequency of K-*ras *mutations, even in this large study, there was not sufficient statistical power to detect statistically significant associations, if they exist. However, the study was conducted to include all cases of relevant sinonasal cancers in Denmark with in a reasonable time frame for conducting interview of the cases. The odds ratios in this study were influenced by some uncertainty, because some calculations were made on groups with less than 10 individuals.

We found an odds ratio for a non significant association between wood dust and K-*ras *mutation on 3.6 [CI = 0.9–15.2] Taking into consideration formaldehyde and smoking reduced the odds ratio (Table 7). However, the mutation frequency in adenocarcinomas were similar to those reported in the studies of Saber et al [20] and Yom et al [21], but it is lower than in a study by Frattini et al [23], who reported of a frequency of 50 % in 17 intestinal type adenocarcinomas from Italian patients. This was not explained by differences in the detection methods used. The sensitivity of the assays in the combination used should be sufficient to identify mutations in the sinonasal tumours. The differences in the prevalence of mutations might be related to differences in the type of exposures. For example hardwoods are used more frequently in Italy (55% of the wood consumed) compared to Northern European countries (8–21% of the wood consumed) [29].

The most frequently found mutation in this study was the GGT^GLY ^→ GAT^ASP ^transitions, which were found in 3 out of 5 mutations. The GGT→GAT transition has been associated with a bad prognosis in studies of lung adenocarcinomas [30,31]. It is also the most common among the previously published K-*ras *codon 12 and 13 mutations in sinonasal cancers. In the study by Saber et al [20], two out of four mutations were GAT transitions, and both were in wood dust exposed patients. In the study by Frattini et al [23], nine mutations were identified. Eight of these were GAT transitions, three of which were from wood dust exposed patients. The remaining one, a GGT^GLY ^→ AGT^SER^, was from a wood dust exposed patient as well. In the study by Perez et al [19] one mutation in the K-*ras *gene, a GAT transition from a wood dust exposed patient was identified.

Summarising mutation data from the present study and previously published ones, it appears that cancers harbouring K-*ras *mutations represent a small fraction of the sinonasal adenocarcinomas. The mutations were primarily located (16/20) at codon 12, and 85% of them were G→A transitions (17/20). All though the number is small, the high frequency of G→A transitions may suggest an environmental component involved in this subgroup of sinonasal cancers. In acute myeloid leukaemia increased frequency of activated ras genes identified a subgroup associated with occupational or chemical exposure [32].

The G→A transitions are typically produced by alkylating agents in experimental systems, consistent with O^6^-methyl-guanine-misparing [33,34]. In humans this transition has been identified in human liver tumours after exposure to vinyl chloride [35,36]. The G→A base transitions in K-*ras *are also very frequently identified in rodent lung neoplasms after long-term exposures to N-nitroso compounds [37]. In tobacco-related lung adenocarcinomas of humans G→T transversions are the most frequent mutations, but G→A transitions are second most frequent [38,39] and most frequent among non smokers [15]. After chronic inhalation to mainstream cigarette smoke in rats the two identified mutations were G→A transitions and G→T transversions [40].

The combination of a G→A transition and exposure to wood dust was present among 60% (9/15) of the G→A mutations where the patient's occupational exposure was known. The frequency of G→A transitions did not differ between wood dust exposed patients and non-exposed (Fisher exact, P = 0.25) However, genotoxic effects have been detected in several studies of healthy wood workers [41-43] and wood dusts were tested positive in the comet assay in the human lung epithelial cell line A549 before an the inflammatory reaction was evoked [44,43].

In addition, in the present study, the two patients with G→A transitions were both smokers. In the study by Saber et al [20], two of the three patients with G→A transitions were smokers. Since exposure to tobacco smoke carcinogens is also associated with G→A transitions, smoking, including involuntary smoking, may also play a role in the carcinogenesis of this subpopulation of sinonasal cancers. To determine the mechanisms behind the carcinogenesis induced by a potential synergistic effects of smoking and wood dust in this subpopulation of sinonasal cancers more studies are needed. Because of the difficulty in achieving statistical power in molecular epidemiology studies of this type cancer, due to the rarity of both sinonasal cancer and K-*ras *mutations, it might be more effective to study the mutagenesis of wood dust in experimental studies.

## Conclusion

We sought to investigate whether exposure to wood dust was associated with a specific mutational pattern in the K-*ras *gene in a large, representative study population. We therefore examined all incident cases of sinonasal cancer in Denmark 1991–2001. Wood dust exposure occurred in 23% of our patients and was associated with a 21-fold [CI = 8.0–55.0] increase in the risk for having an adenocarcinoma than a squamous cell carcinoma compared to not being exposed. The frequency of K-*ras *mutations in adenocarcinoma (13%) was in the range reported in earlier studies, whereas the frequency was very low in the squamous cell carcinoma (1%). Collectively, taking into account the mutations we detected our and the ones in sinonasal cancers that have been reported before, the GGT^GLY ^→ GAT^ASP ^transition is the most common mutation after exposure to wood dust. In conclusion, mutational activation of K-*ras *in SNC is restricted to a subgroup of the adenocarcinoma histology. However, since most of the mutation positive cases were smokers, active or passive exposure to cigarette smoke, may also be involved. The observed mutation types are in keeping with this. Overall, the study suggests a limited role for K-*ras *mutations in development of sinonasal cancer.

## Competing interests

The author(s) declare that they have no competing interests.

## Authors' contributions

JB participated in study design, collected the study material, performed the molecular analyses, data analysis, and drafted this manuscript. JH conceived the epidemiological part of the study. JH and JB statistically analysed the data. TS, MD and AA selected the tissue blocks. TS and MD performed the pathological review of the blocks. HW contributed to the pathological diagnoses and organized the immunohistochemical stainings. VS assessed the wood dust exposure. HWA, RH, DL and KHP conceived the study. UV and HWA participated in the study design and co-wrote the manuscript. All authors read and approved the final manuscript.

## Pre-publication history

The pre-publication history for this paper can be accessed here:


